# Association between oxidative balance score and all-cause and cancer-specific mortality among cancer survivors

**DOI:** 10.3389/fimmu.2025.1541675

**Published:** 2025-03-20

**Authors:** Qingmei Gao, Xinfang Zhu, Mengke Chen, Rong Xia, Qi Zhang

**Affiliations:** Department of Blood Transfusion, Huashan Hospital, Fudan University, Shanghai, China

**Keywords:** oxidative balance score, all-cause mortality, cancer mortality, cancer survivors, diet, lifestyle

## Abstract

**Background:**

The Oxidative Balance Score (OBS) represents a novel metric for assessing systemic oxidative stress, where elevated scores reflect increased antioxidant exposure. This study aims to explore the association between OBS and all-cause and cancer-specific mortality among cancer survivors.

**Methods:**

An observational cohort study was conducted involving 4099 cancer survivors, utilizing data obtained from the National Health and Nutrition Examination Survey (NHANES) covering the years 1999 to 2018. The endpoints were established by cross-referencing data with the National Death Index (NDI). The OBS was developed based on dietary and lifestyle factors. Cox proportional hazards regression models were employed to examine the relationship between OBS and mortality risks. Restricted cubic spline was utilized to evaluate whether OBS exhibited a nonlinear association with the risk of death. Furthermore, Kaplan-Meier survival curves were generated to assess cumulative survival differences across various OBS outcomes.

**Results:**

Over an average follow-up of 84.00 months, 1481 (26.29%) participants died, including 484 (8.9%) who died from cancer. In the fully adjusted model, multivariable Cox regression revealed that each unit increase in OBS was linked to a 1.8% decrease in all-cause mortality risk (HR 0.982, 95%CI 0.972-0.991) and a 2.6% decrease in cancer-specific mortality risk (HR 0.974, 95%CI 0.958-0.991). In the context of all-cause mortality, the risk of death was found to be significantly lower in quartiles Q2, Q3 and Q4 when compared to the OBS in quartile Q1. The hazard ratios (HRs) and 95% confidence intervals (CIs) for Q2, Q3 and Q4 were as follows: Q2 (HR 0.833, 95%CI 0.707-0.981), Q3 (HR 0.789, 95%CI 0.650-0.958) and Q4 (HR 0.699, 95%CI 0.579-0.844). Regarding cancer-specific mortality, the HRs and 95%CIs for Q2, Q3 and Q4 in comparison to Q1 were as follows: Q2 (HR 0.663, 95%CI 0.505-0.869), Q3 (HR 0.688, 95%CI 0.488-0.969) and Q4 (HR 0.595, 95%CI 0.435-0.815). Similar associations were noted when the dietary and lifestyle components of the OBS were analyzed separately.

**Conclusion:**

The findings indicate that higher levels of OBS are associated with a decrease in all-cause and cancer-specific mortality among cancer survivors. Our findings may contribute to the refinement of lifestyle intervention recommendations for this population.

## 1 Introduction

Cancer remains one of the most pressing public health challenges globally, marked by rising incidence and mortality rates. According to the latest estimates from the International Agency for Research on Cancer (IARC), nearly 20 million new cases of cancer (including non-melanoma skin cancer [NMSC]) were reported in 2022, resulting in approximately 9.7 million cancer-related deaths (including NMSC). It is estimated that one in five individuals, regardless of gender, will get cancer during their lifetime, with approximately one in nine men and one in twelve women succumbing to the disease (1). Research has demonstrated that dietary and lifestyle factors play a significant role in the occurrence and progression of cancer. In recent years, numerous studies have focused on the positive effects of modifiable dietary and lifestyle factors in cancer prevention and the improvement of cancer prognosis (2–4).

The emergence of various pathological and physiological conditions, including cancer, is frequently linked to excessive cellular oxidative stress. Cells maintain redox homeostasis to prevent oxidative damage to DNA, proteins, and lipids (5). The disruption of the equilibrium between pro-oxidants and antioxidants, commonly referred to as oxidative stress, has been recognized as a significant pathogenic and pathophysiological factor in numerous chronic diseases, which are leading contributors to mortality (6, 7). The Oxidative Balance Score (OBS) is a metric designed to quantify the overall exposure to oxidants and antioxidants within an individual’s diet and lifestyle. A higher OBS indicates a predominant exposure to an excess of antioxidants (8). In contrast to research that concentrates on individual nutrients, the OBS offers a substantial advantage by integrating multiple oxidants and antioxidants, thereby providing a more comprehensive assessment of overall oxidative stress. Empirical evidence suggests that the OBS is an effective tool for evaluating oxidative stress status (9, 10).

To date, there have been limited epidemiological studies investigating the association between OBS and cancer prognosis (11). Consequently, the present study seeks to examine the relationship between OBS and the prognosis of cancer survivors, utilizing data from the National Health and Nutrition Examination Survey.

## 2 Materials and methods

### 2.1 Study population

The National Center for Health Statistics (NCHS), a component of the Centers for Disease Control and Prevention (CDC), initiated multiple cycles of the National Health and Nutrition Examination Survey (NHANES) in the United States beginning in 1999. This study utilized a retrospective observational cohort design, analyzing data obtained from NHANES spanning the years 1999 to 2018. NHANES employs a complex, stratified, multi-stage probability cluster design to conduct a nationally representative survey of the health and nutritional status of the non-institutionalized civilian population in the United States. The survey provides comprehensive information regarding its methodologies and analytical guidelines (12). Data concerning nutrition and health status are collected through a series of household interviews, physical examinations, and laboratory measurements.

Furthermore, the National Center for Health Statistics (NCHS) has established connections between numerous demographic surveys and death certificate data obtained from the National Death Index (NDI). The data have been meticulously processed to reduce the risk of subject identification, and a publicly accessible version of the follow-up data concerning mortality rates for adult participants, spanning from the date of survey participation until December 31, 2019, has been made available.

**Figure 1:**
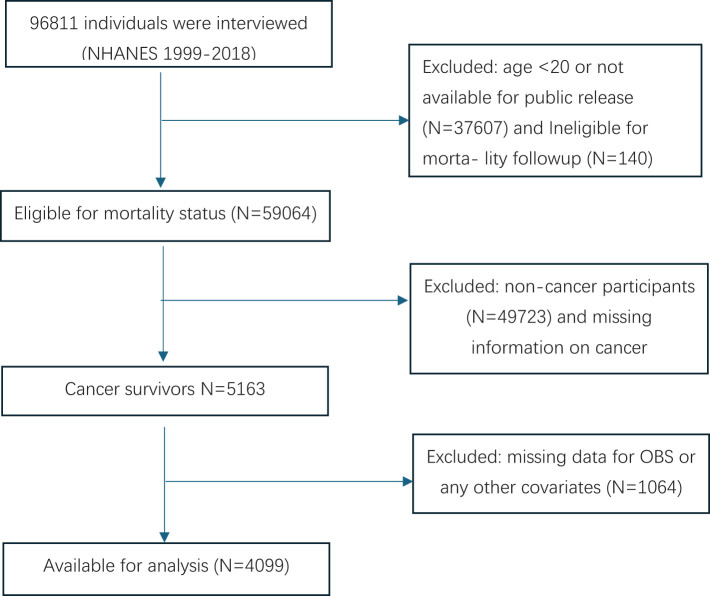
Flowchart for selecting analyzed participants.

Initially, a total of 96,811 participants were included in the study. However, we excluded participants under the age of 20 whose data were not publicly available (N=37,607) and those who did not meet the mortality follow-up criteria (N=140). We also excluded individuals without information from the malignant tumor questionnaire (N=4,718) and based on the cancer questionnaire, we excluded those who denied having cancer or malignancies (N=49,723). Additionally, individuals lacking dietary and lifestyle data necessary for calculating the oxidative balance score and any specified study covariates (N=1,064) were also excluded. The final cohort included 4,099 cancer survivors (see **Figure 1**).

### 2.2 Exposure variable: oxidative balance score

The calculation of the Oxidative Balance Score (OBS) encompasses 16 dietary nutrients and 4 lifestyle factors, which consist of 15 antioxidant components and 5 pro-oxidant components (13). A majority of components previously utilized for the calculation of the Oxidative Balance Score(OBS) have been identified, while six additional components have been newly selected based on their availability in the data and their correlation with oxidative stress (OS). These components include riboflavin, niacin, vitamin B6, vitamin B12, magnesium, and copper (8). These 16 nutrients are obtained from the initial dietary recall interview. The 4 lifestyle factors considered are physical activity, body mass index (BMI), alcohol consumption, and smoking, with smoking levels assessed through cotinine measurements. The 5 pro-oxidant components identified are total fat, iron, BMI, alcohol consumption, and cotinine, while the remaining factors are categorized as antioxidant components. The components of oxidative balance score (OBS) are categorized into four distinct groups: (1) dietary antioxidants, which include fiber, β-carotene, riboflavin, niacin, vitamin B6, total folate, vitamin B12, vitamin C, vitamin E, calcium, magnesium, zinc, copper, and selenium; (2) dietary pro-oxidants, comprising total fat and iron; (3) lifestyle antioxidants, represented by physical activity; and (4) lifestyle pro-oxidants, which encompass alcohol, smoking, and BMI.

According to the existing literature (13), alcohol consumption is classified into three categories: non-drinkers, light to moderate drinkers (defined as women consuming 0-15 grams per day and men consuming 0-30 grams per day), and heavy drinkers (defined as women consuming 15 grams or more per day and men consuming 30 grams or more per day). These categories are assigned scores of 2, 1, and 0, respectively (13). The levels of physical activity (PA) were assessed utilizing the NHANES Physical Activity Questionnaire (PAQ).In the process of calculating the PA score, it is essential to first gather pertinent data from the Physical Activity Questionnaire (PAQ) (14). Subsequently, the PAQ data will be converted into Metabolic Equivalent (MET) using the following formula: Physical Activity (PA) in MET-minutes per week (MET-min/wk) = MET × weekly frequency of each physical activity (PA) × duration. Due to the variations in the NHANES Physical Activity Questionnaire (PAQ) across different cycles, the physical activity calculations were modified to align with the respective survey year. From 1999 to 2006, PA was classified into categories such as walking or bicycling, home or yard tasks, muscle-strengthening activities, and screen time (e.g., watching television or using a computer). In contrast, from 2007 to 2018, PA was organized into two overarching categories: work-related activities, which were further subdivided into moderate-intensity and vigorous-intensity tasks, and leisure activities, encompassing walking, bicycling, and both vigorous- and moderate-intensity recreational activities. In accordance with NHANES recommendations, the metabolic equivalent (MET) score coefficients for both vigorous work-related and leisure time activities were set at 8.0, while the MET score coefficients for moderate work-related and leisure time activities, as well as for walking or bicycling as a mode of transportation, were established at 4.0. We evaluated physical activity levels utilizing MET scores in accordance with the NHANES guidelines. Participants were classified into three distinct categories: 0 points for those engaging in less than 400 MET minutes per week, 1 point for those accumulating between 400 and 1,000 MET minutes per week, and 2 points for individuals exceeding 1,000 MET minutes per week. Other components are evaluated based on quartiles, stratified by gender. For antioxidant components, scores range from 0 to 2, corresponding to the lowest and highest quartiles, respectively. Conversely, the scoring for pro-oxidant components is inverted, with the highest quartile receiving 0 points and the lowest quartile receiving 2 points. Detailed scoring criteria for each OBS component are provided in **Supplementary Table 1**. The overall Oxidative Balance Score (OBS) is computed by aggregating the scores of each component, resulting in a score range of 3 to 36, where a higher score signifies greater exposure to antioxidants. Furthermore, we categorized the OBS into dietary and lifestyle components based on their sources to investigate their combined and independent effects on the mortality risk among cancer survivors.

### 2.3 Definition of cancer and classification of cancer types

Participants who responded affirmatively to the inquiry, “Have you ever been told by a doctor or other health professional that you had cancer or a malignancy of any kind?” (variable MCQ220)?”. Individuals who answer “yes” were categorized as cancer survivors and were subsequently asked What kind of cancer was it?” and “How old were you when this cancer was first diagnosed?. The variable “years since first cancer diagnosis” was calculated by subtracting the age at which the participant was first diagnosed with cancer from their current age.

### 2.4 Follow-up and outcomes

The follow-up period extends from the date of the interview to the last follow-up date, which is December 31, 2019, or to the date of death, whichever occurs first. The National Death Index (NDI) records furnish information regarding the causes of death for the participants included in the study. The mortality outcomes are classified in accordance with the International Classification of Diseases and Related Health Problems, 10th Revision (ICD-10) codes, which are documented as the primary cause of death. The study endpoints encompass all-cause mortality as well as cancer-related mortality, specifically codes C00 to C97.

### 2.5 Covariates

During the household interviews, demographic data were collected, including age, gender, race and ethnicity (categorized as Hispanic American, non-Hispanic Black, non-Hispanic White, or other), education level (less than high school, high school or equivalent, or college or above), marital status (single or non-single), poverty income ratio (PIR), and medical history concerning diabetes, hypertension, and cardiovascular disease (CVD). The poverty income ratio serves as an index of poverty status, calculated by dividing the total household income by the poverty threshold. In accordance with the analysis guidelines, this ratio is classified into three categories: ≤1.3, 1.3-3.5, and >3.5 (12). The definition of diabetes is based on an affirmative response to any of the following questions:: “Have you ever been told by a doctor that you have diabetes?” or “Are you now taking insulin?” or “Are you now taking diabetes pills to lower your blood sugar?”. Hypertension is defined as a self-reported diagnosis, the use of antihypertensive medication, or meeting any of the following criteria: systolic/diastolic blood pressure of ≥140/90 mmHg. Cardiovascular disease (CVD) is defined as a self-reported diagnosis of any of the following conditions: stroke, angina pectoris, myocardial infarction, coronary heart disease, or heart failure.

### 2.6 Statistical analysis

In our analysis, the complex sampling design of the National Health and Nutrition Examination Survey (NHANES) incorporated sample weights, clustering, and stratification, which are essential for the appropriate analysis of NHANES data (15). Baseline characteristics are represented by the quartiles of the observed variable (OBS). Normally distributed data are presented as mean ± standard error (SE), while non-normally distributed data are expressed as median (interquartile range), and categorical variables are reported as counts (percentages). The Kaplan-Meier method was employed to plot survival curves associated with OBS. Multivariable Cox regression models (Model 1, Model 2, and Model 3) were utilized to evaluate the relationship between OBS and both all-cause mortality and cancer mortality. Model 1 was not adjusted for any covariates. Model 2 was adjusted for baseline demographic variables, which included age (measured in continuous years), gender, race, and ethnicity (categorized as Hispanic American, non-Hispanic Black, non-Hispanic White, or other). Furthermore, Model 3 included additional adjustments for social factors, such as education level, marital status, and poverty income ratio, as well as for chronic diseases, which encompassed diabetes, hypertension, and cardiovascular diseases(CVD). The log-rank test and Kaplan-Meier (K-M) survival analysis were employed to investigate differences in survival probabilities. To visualize the association between OBS and both all-cause mortality and cancer mortality among cancer survivors, a restricted cubic spline (RCS) with three knots was constructed based on the fully adjusted Cox model. Additionally, we conducted a series of sensitivity analyses to evaluate the robustness of our findings. Initially, we excluded participants who succumbed to cancer within 2 years of diagnosis to mitigate the potential for reverse causality. Subsequently, we incorporated an additional adjustment covariate: the year of diagnosis. Finally, we performed independent stratified analyses within specific subgroups categorized by age, sex, race, education, poverty-to-income ratio, hypertension, diabetes, and CVD to ascertain whether the associations between observational variables and all-cause mortality, as well as cancer mortality, varied across these subgroups for each covariate category.

All statistical analyses were performed utilizing R version 4.4.1. A two-sided p-value of less than 0.05 was deemed statistically significant.

## 3 Results

### 3.1 Baseline characteristics of participants

**Table 1** presents the baseline characteristics of participants categorized by OBS quartiles. The study comprised a total of 4,099 cancer survivors. The mean age of participants was 62.34 years (± 0.36), with non-Hispanic whites constituting the predominant demographic within the study population. No statistically significant differences were observed in age and gender across the groups (P all >.05). In comparison to participants in the lowest OBS quartile (Q1), those in the highest OBS quartile (Q4) were more likely to be non-Hispanic white, not single, possess a higher poverty income level, and have no prior history of diabetes, hypertension, or cardiovascular disease. Additional data on cancer survivors classified by cancer type and OBS quartiles can be found in the **Supplementary Materials**, specifically in **Supplementary Table 2**.

**Table 1:** Baseline characteristics of cancer survivors.

Characteristic	Total (n=4099)	Q1 (n=1146)	Q2 (n=1068)	Q3 (n=863)	Q4 (n=1022)	P value
Age (years), mean (SE)	62.34 (0.36)	61.54 (0.71)	62.12 (0.54)	63.31 (0.62)	62.49 (0.61)	0.21
Gender,n(%)						0.32
Female	2126 (56.75)	582 (56.44)	554 (56.17)	442 (53.92)	548 (59.71)	
Male	1973 (43.25)	564 (43.56)	514 (43.83)	421 (46.08)	474 (40.29)	
Race and ethnicity,n(%)						< 0.0001
Mexican American	265 (2.18)	78 (2.30)	74 (1.87)	54 (2.51)	59 (2.12)	
Non-Hispanic Black	564 (5.38)	237 (9.16)	136 (5.03)	93 (4.49)	98 (3.15)	
Non-Hispanic White	2910 (87.00)	737 (82.88)	759 (86.70)	629 (86.50)	785 (91.17)	
Other	360 (5.44)	94 (5.66)	99 (6.39)	87 (6.49)	80 (3.55)	
Married status,n(%)						0.01
non-single	2502 (66.17)	658 (60.61)	649 (65.22)	541 (67.32)	654 (70.93)	
single	1597 (33.83)	488 (39.39)	419 (34.78)	322 (32.68)	368 (29.07)	
Education,n(%)						< 0.0001
Below high school	396 (4.93)	160 (7.99)	120 (5.91)	64 (3.93)	52 (2.16)	
College graduate or above	1017 (31.81)	150 (17.59)	262 (32.05)	232 (32.64)	373 (43.05)	
High school/Some college	2052 (47.93)	674 (58.41)	510 (46.16)	433 (48.20)	435 (40.43)	
Some College or AA degree	634 (15.34)	162 (16.01)	176 (15.88)	134 (15.23)	162 (14.35)	
Poverty to income ratio n(%)						< 0.0001
<=1.3	988 (16.64)	391 (25.81)	264 (17.83)	159 (11.50)	174 (11.64)	
>=3.5	1436 (47.36)	272 (32.63)	359 (46.08)	313 (48.86)	492 (59.96)	
1.3-3.5	1675 (36.00)	483 (41.57)	445 (36.09)	391 (39.64)	356 (28.40)	
Status						< 0.0001
survival	2618 (73.71)	657 (65.43)	672 (74.11)	553 (73.10)	736 (80.85)	
death	1481 (26.29)	489 (34.57)	396 (25.89)	310 (26.90)	286 (19.15)	
Age group, n(%)						0.18
>=60	2971 (61.20)	829 (58.42)	780 (60.38)	640 (65.30)	722 (61.20)	
20-59	1128 (38.80)	317 (41.58)	288 (39.62)	223 (34.70)	300 (38.80)	
Hypertension,n(%)						< 0.001
no	1481 (42.26)	353 (36.97)	363 (39.00)	327 (43.62)	438 (48.79)	
yes	2618 (57.74)	793 (63.03)	705 (61.00)	536 (56.38)	584 (51.21)	
Diabetes,n(%)						0.001
DM	840 (16.42)	285 (19.76)	215 (17.08)	186 (17.52)	154 (12.15)	
no	3259 (83.58)	861 (80.24)	853 (82.92)	677 (82.48)	868 (87.85)	
CVD,n(%)						< 0.0001
no	3079 (80.14)	779 (72.21)	794 (78.67)	677 (82.67)	829 (86.35)	
yes	1020 (19.86)	367 (27.79)	274 (21.33)	186 (17.33)	193 (13.65)	

### 3.2 The association between obesity and all-cause mortality as well as cancer mortality

The median follow-up duration was 84.00 months (range: 45.00 to 140.00 months), during which a total of 1,481 individuals (26.29%) succumbed, including 484 individuals who died from cancer. **Table 2** illustrates the association between oxidative Balance Score (OBS), encompassing both dietary and lifestyle factors, and all-cause as well as cancer-specific mortality among cancer survivors. In the unadjusted model (Model 1), an increase in OBS was significantly correlated with a decrease in the risk of all-cause mortality (hazard ratio [HR]0.972, 95% confidence interval [CI] 0.963–0.982, p < 0.0001). When comparing the lowest quartile of OBS (Q1) to the highest quartile (Q4), the latter was associated with a 45.3% reduction in the risk of all-cause mortality (HR 0.547, 95% CI 0.452-0.663, p for trend <0.0001). In Model 2, after adjusting for gender, age, and race, each unit increase in OBS corresponded to a 3.3% reduction in the risk of all-cause mortality (HR 0.967, 95% CI 0.958–0.975, p < 0.0001). In this adjusted model, participants in the OBS Q4 group exhibited a 46.5% lower risk of all-cause mortality compared to the Q1 group (HR 0.535, 95% CI 0.450–0.636, p for trend <0.0001). In the fully adjusted model (Model 3), each unit increase in OBS was associated with a 1.8% reduction in the risk of all-cause mortality (HR 0.982, 95% CI 0.972–0.991, p < 0.0001). When comparing the Q4 group to the Q1 group, the risk of all-cause mortality was reduced by 30.1% (HR 0.699, 95% CI 0.579–0.844, p for trend <0.001). Furthermore, in Model 3, both dietary OBS and lifestyle OBS demonstrated a negative correlation with the risk of all-cause mortality among cancer survivors (p = 0.006 and p < 0.0001, respectively). In this model, compared to Q1, the dietary OBS and lifestyle OBS in Q4 were associated with a 24.5% reduction in all-cause mortality risk (HR 0.755, 95% CI 0.637–0.896, p for trend <0.001) and a 41.7% reduction in all-cause mortality risk (HR 0.583, 95% CI 0.477–0.713, p for trend <0.0001), respectively.

**Table 2:** Multivariable Cox regression models analysis of the relationship between OBS and mortality among cancer survivors in NHANES 1999–2018.

Exposure	Model 1	Model 2	Model 3
	HR (95% CI) P value	HR (95% CI) P value	HR (95% CI) P value
All causes			
OBS	0.972 (0.963,0.982)<0.0001	0.967 (0.958,0.975)<0.0001	0.982 (0.972,0.991)<0.001
OBS (Quartile)			
Q1	Reference	Reference	Reference
Q2	0.733 (0.606,0.886) 0.001	0.724 (0.616,0.851)<0.0001	0.833 (0.707,0.981)0.029
Q3	0.761 (0.613,0.944)0.013	0.684 (0.563,0.831)<0.001	0.789 (0.650,0.958)0.017
Q4	0.547 (0.452,0.663)<0.0001	0.535 (0.450,0.636)<0.0001	0.699 (0.579,0.844)<0.001
P for Trend	<0.0001	<0.0001	<0.001
OBS.DIETARY	0.973 (0.963,0.984)<0.0001	0.973 (0.964,0.983)<0.0001	0.986 (0.976,0.996)0.006
OBS.DIETARY (Quartile)			
Q1	Reference	Reference	Reference
Q2	0.738 (0.610,0.891)0.002	0.765 (0.634,0.923)0.005	0.870 (0.721,1.049)0.145
Q3	0.755 (0.619,0.920)0.005	0.680 (0.566,0.817)<0.0001	0.784 (0.653,0.941)0.009
Q4	0.594 (0.497,0.710)<0.0001	0.612 (0.519,0.721)<0.0001	0.755 (0.637,0.896)0.001
P for Trend	<0.0001	<0.0001	<0.001
OBS.LIFESTYLE	0.907 (0.866,0.950)<0.0001	0.835 (0.799,0.873)<0.0001	0.887 (0.844,0.932)<0.0001
OBS.LIFESTYLE (Quartile)			
Q1	Reference	Reference	Reference
Q2	0.822 (0.693,0.976)0.025	0.718 (0.613,0.841)<0.0001	0.809 (0.689,0.949)0.009
Q3	0.833 (0.679,1.021)0.078	0.698 (0.582,0.837)<0.001	0.800 (0.662,0.967)0.021
Q4	0.596 (0.474,0.749)<0.0001	0.445 (0.366,0.541)<0.0001	0.583 (0.477,0.713)<0.0001
P for Trend	<0.0001	<0.0001	<0.0001
Cancer			
OBS	0.968 (0.950,0.986)<0.001	0.963 (0.946,0.981)<0.0001	0.974 (0.958,0.991)0.003
OBS (Quartile)			
Q1	Reference	Reference	Reference
Q2	0.619 (0.461,0.831)0.001	0.608 (0.461,0.803)<0.001	0.663 (0.505,0.869)0.003
Q3	0.677 (0.483,0.949)0.023	0.609 (0.433,0.855)0.004	0.688 (0.488,0.969)0.032
Q4	0.520 (0.368,0.735)<0.001	0.492 (0.356,0.680)<0.0001	0.595 (0.435,0.815)0.001
P for Trend	<0.001	<0.0001	0.003
OBS.DIETARY	0.971 (0.952,0.990)0.004	0.970 (0.952,0.989)0.002	0.981 (0.963,0.999)0.035
OBS.DIETARY (Quartile)			
Q1	Reference	Reference	Reference
Q2	0.636 (0.476,0.849)0.002	0.656 (0.495,0.871)0.003	0.733 (0.557,0.966)0.027
Q3	0.562 (0.409,0.772)<0.001	0.513 (0.371,0.710)<0.0001	0.583 (0.421,0.808)0.001
Q4	0.594 (0.421,0.840)0.003	0.596 (0.429,0.827)0.002	0.699 (0.505,0.967)0.030
P for Trend	0.004	0.002	0.019
OBS.LIFESTYLE	0.864 (0.804,0.928)<0.0001	0.802 (0.749,0.859)<0.0001	0.834 (0.775,0.896)<0.0001
OBS.LIFESTYLE (Quartile)			
Q1	Reference	Reference	Reference
Q2	0.800 (0.593,1.080)0.145	0.698 (0.516,0.945)0.020	0.758 (0.559,1.030)0.076
Q3	0.726 (0.520,1.015)0.061	0.636 (0.466,0.869)0.005	0.681 (0.498,0.931)0.016
Q4	0.457 (0.314,0.664)<0.0001	0.343 (0.240,0.490)<0.0001	0.414 (0.287,0.597)<0.0001
P for Trend	<0.0001	<0.0001	<0.0001

Model 1, unadjusted;

Model 2, adjusted for age, gender, race;

Model 3, adjusted for age, gender, race, education, marital status, poverty to income ratio, hypertension, diabetes, and CVD.

**Table 2** presents the association between OBS and cancer mortality. In the unadjusted model (Model 1), a significant reduction in cancer mortality risk was observed with increasing OBS (HR 0.968, 95% CI 0.950, 0.986, p < 0.001). When comparing the OBS quartile 1 (Q1) group to the quartile 4 (Q4) group, the cancer mortality risk decreased by 48.0% (HR 0.520, 95% CI 0.368, 0.735, p for trend < 0.001). In Model 2, after adjusting for gender, age, and race, each unit increase in OBS was associated with a 6.7% reduction in cancer mortality risk (HR 0.963, 95% CI 0.946, 0.981, p < 0.0001). Participants in the Q4 group exhibited a 50.8% lower cancer mortality risk compared to the Q1 group (HR 0.492, 95% CI 0.356, 0.680, p for trend < 0.0001). In the fully adjusted model (Model 3), each unit increase in OBS corresponded to a 2.6% decrease in cancer mortality risk (HR 0.974, 95% CI 0.958, 0.991, p = 0.003). The cancer mortality risk in the OBS Q4 group was reduced by 40.5% relative to the Q1 group (HR 0.595, 95% CI 0.435, 0.815, p for trend = 0.003). Furthermore, in Model 3, both dietary OBS and lifestyle OBS demonstrated a negative correlation with cancer mortality risk among cancer survivors (p = 0.035 and p < 0.0001, respectively). Specifically, compared to participants in the Q1 group, those in the Q4 group exhibited a 30.1% lower mortality risk associated with dietary OBS (HR 0.699, 95% CI 0.505, 0.967, p for trend = 0.019) and a 58.6% lower cancer mortality risk associated with lifestyle OBS (HR 0.414, 95% CI 0.287, 0.597, p for trend < 0.0001).

**Figure 2:**
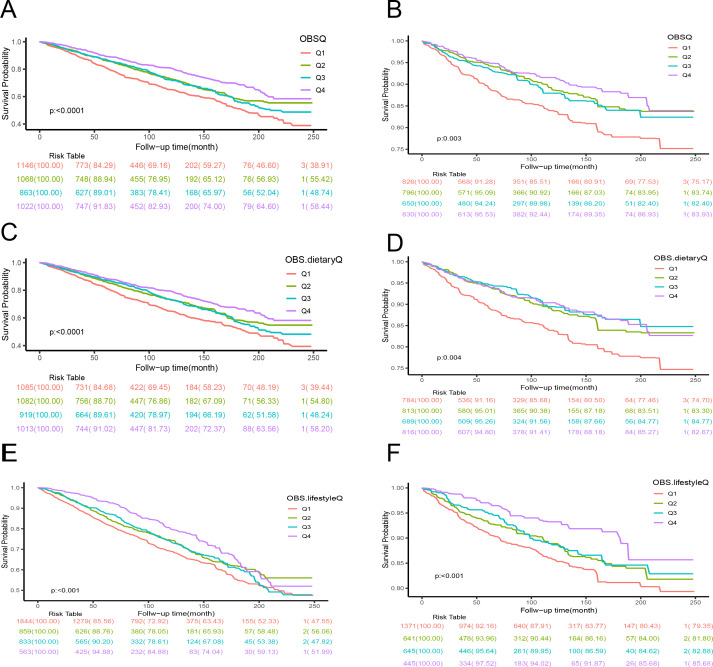
The Kaplan-Meier analyses for mortality across the four groups are presented as follows: **(A)** Kaplan-Meier curve illustrating the relationship between observational (OBS) data and all-cause mortality; **(B)** Kaplan-Meier curve depicting the association between OBS data and cancer-specific mortality; **(C)** Kaplan-Meier curve representing the correlation between dietary OBS and all-cause mortality rate; **(D)** Kaplan-Meier curve showing the link between dietary OBS and cancer-specific mortality; **(E)** Kaplan-Meier curve for lifestyle OBS and all-cause mortality rate; **(F)** Kaplan-Meier curve for lifestyle OBS and cancer-specific mortality.

Kaplan-Meier (K-M) survival analysis, as illustrated in **Figure 2**, indicated significant differences in all-cause mortality and cancer mortality across the four groups (all P < 0.05). Participants in the highest quartile (Q4) of the observational variable (OBS) exhibited the lowest mortality rate, whereas those in the lowest quartile (Q1) demonstrated the highest mortality rate.

**Figure 3:**
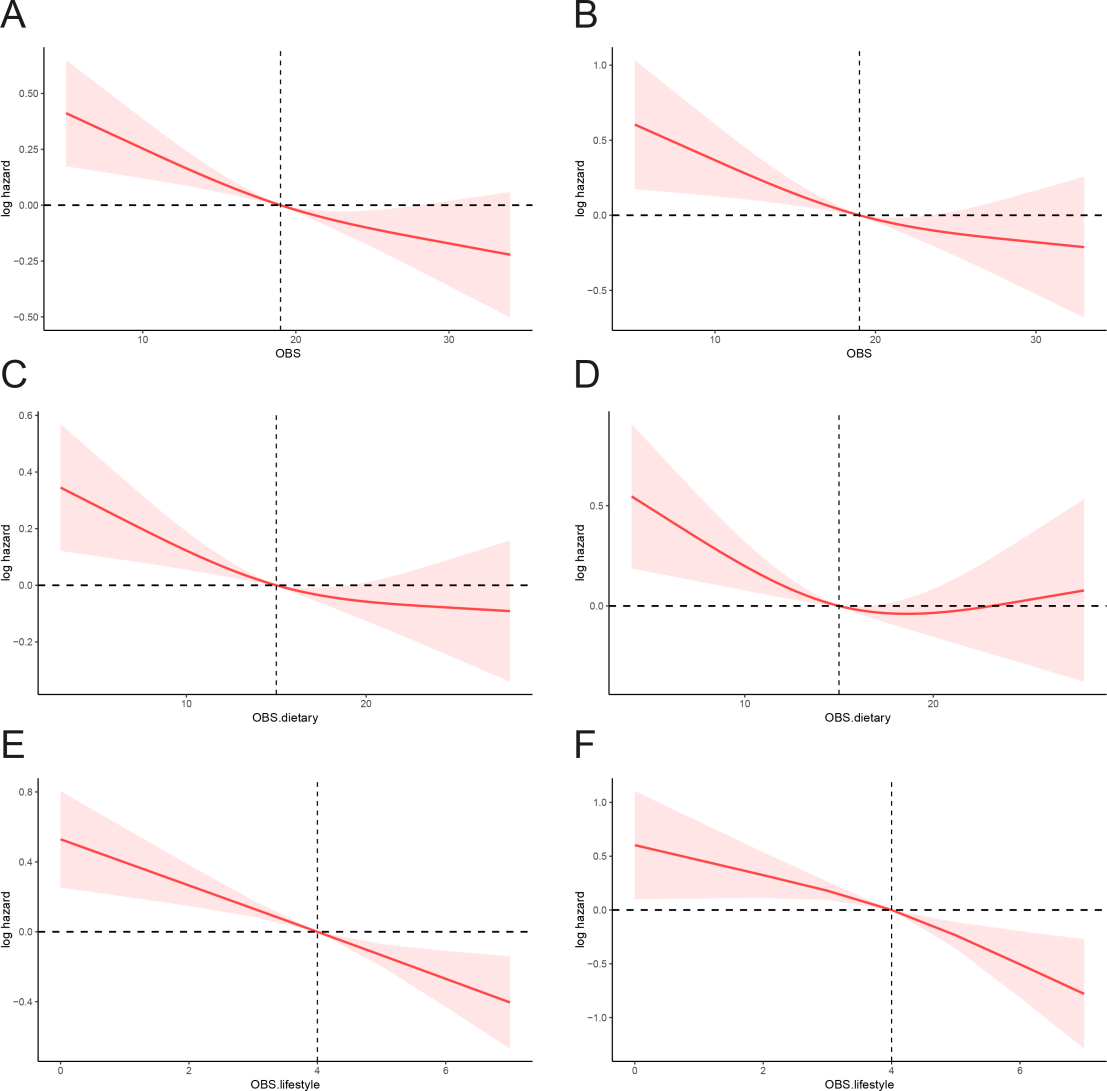
RCS analyses for mortality: **(A)** OBS and all-cause mortality; **(B)** OBS and cancer-specific mortality; **(C)** Dietary OBS and all-cause mortality; **(D)** Dietary OBS and cancer-specific mortality; **(E)** Lifestyle OBS and all-cause mortality; **(F)** Lifestyle OBS and cancer-specific mortality.

We also performed a restricted cubic spline (RCS) analysis utilizing the fully adjusted Cox regression model (Model 3). The results indicated a negative correlation between OBS and both all-cause mortality and cancer mortality among cancer survivors (see **Figure 3**).

### 3.3 Subgroup analysis

A stratified analysis was performed to evaluate the robustness of the regression results concerning the relationship between OBS and mortality among various subgroups of cancer survivors (see **Table 3**). The findings suggest that, in the majority of subgroups, the relationship between OBS and mortality is consistent, exhibiting a negative correlation that is statistically significant. Furthermore, no significant interactions were identified between age, gender, race, education level, marital status, poverty income ratio (PIR), hypertension, diabetes, cardiovascular disease (CVD) history, and OBS (P for interaction>0.05). To mitigate the potential for reverse causality, we excluded participants who succumbed to cancer within two years of their diagnosis. The results remained statistically significant (**Supplementary Table 3**). Additionally, further adjustments for the number of years since diagnosis did not yield a significant alteration in the results (**Supplementary Table 4**).

**Table 3:** Stratified analysis of the relationship between OBS and mortality among cancer survivors in NHANES 1999–2018.

	All-cause mortality	Cancer-specific mortality
	HR (95% CI) P value	HR (95% CI) P value
Age		
<60	0.972 (0.940,1.005)0.100	0.959 (0.916,1.003)0.070
>=60	0.984 (0.975,0.994)0.001	0.981 (0.963,0.999)0.036
P for interaction	0.09	0.113
Gender		
Female	0.978 (0.965,0.990)<0.001	0.973 (0.946,1.001)0.057
Male	0.986 (0.974,0.999)0.036	0.974 (0.955,0.994)0.011
P for interaction	0.285	0.952
Race and ethnicity		
Non-Hispanic White	0.980 (0.969,0.990)<0.001	0.969 (0.951,0.988)0.001
Mexican American	0.999 (0.962,1.038)0.970	0.976 (0.921, 1.034)0.410
Non-Hispanic Black	0.996 (0.969,1.023)0.769	0.996 (0.957,1.036)0.836
Other	0.966 (0.921,1.014)0.160	1.003 (0.948, 1.061)0.912
p for interaction	0.304	0.159
Education		
High school/Some college	0.987 (0.974,1.000)0.043	0.975 (0.952,0.998)0.035
Below high school	0.968 (0.943,0.994)0.016	0.966 (0.914,1.020)0.214
Some College or AA degree	0.971 (0.949,0.994)0.014	0.958 (0.924, 0.994)0.021
College graduate or above	0.985 (0.966,1.004)0.110	0.984 (0.950, 1.019)0.364
p for interaction	0.484	0.741
Poverty to income ratio		
<=1.3	0.989 (0.974,1.004)0.139	0.968 (0.936,1.000)0.051
>=3.5	0.979 (0.959,1.000)0.047	0.961 (0.932, 0.991)0.011
1.3-3.5	0.972 (0.955,0.990)0.002	0.990 (0.965,1.015)0.413
p for interaction	0.448	0.405
Hypertension		
no	0.979 (0.961,0.998)0.032	0.965 (0.936,0.994)0.020
yes	0.983 (0.972,0.993)0.001	0.979 (0.959,0.998)0.032
p for interaction	0.329	0.381
Diabetes		
no	0.979 (0.969,0.989)<0.0001	0.972 (0.955,0.990)0.003
DM	0.995 (0.974,1.017)0.662	0.981 (0.932,1.032)0.457
p for interaction	0.08	0.799
CVD		
yes	0.978 (0.962,0.994)0.007	0.959 (0.928,0.991)0.013
no	0.985 (0.973,0.997)0.014	0.979 (0.960,0.998)0.029
p for interaction	0.647	0.299

A hierarchical analysis was performed using Model 3. In each instance, the model did not include the hierarchical variables themselves.

## 4 Discussion

This study represents a pioneering effort to elucidate the influence of modifiable dietary and lifestyle factors, specifically pro-antioxidants and antioxidants on the survival prognosis of cancer survivors. The research employs a comprehensive measure of oxidative stress known as the Oxidative Balance Score (OBS). Through the analysis of nationally representative data from the United States collected over the past two decades, we found that elevated OBS scores correlated with a reduced risk of all-cause mortality and cancer-specific mortality. Furthermore, similar associations were observed for the dietary and lifestyle components of the OBS when examined independently. The findings presented above suggest that diets and lifestyles characterized by elevated levels of antioxidants, such as those abundant in antioxidant fiber,β-carotene, riboflavin, niacin, vitamin B6, total folate, vitamin B12, vitamin C, vitamin E, calcium, magnesium, zinc, copper, and selenium, while concurrently low in pro-oxidant fats and iron, combined with adherence to a physical activity regimen, reduced alcohol consumption, and avoidance of smoking, are associated with a decreased risk of all-cause mortality among cancer survivors, as well as a reduced risk of cancer-specific mortality.

In recent years, the relationship between oxidative stress and cancer has attracted considerable interest within the scientific community. Oxidative stress is intricately linked to tumor development and can affect the behavior of tumor cells through complex regulatory networks, including mitochondrial stress, endoplasmic reticulum stress, and ferritin deposition (16). ROS has the capacity to directly damage DNA, resulting in various forms of DNA damage, such as strand breaks. This damage can alter the expression levels of essential genes associated with proto-oncogenes, oncogenes, and DNA damage repair mechanisms, thereby facilitating tumorigenesis (17, 18). Furthermore, ROS can induce mutagenic break repair and activate the SOS response by damaging DNA bases, which interrupts replication and triggers a critical transition from high-fidelity to error-prone DNA polymerases, ultimately leading to an increase in carcinogenic mutations (19, 20). Research indicates that ROS can influence tumorigenesis and cellular transformation by oxidizing cysteine residues, which subsequently activate the three most prevalent oncogenic switch genes in human cancers: HRAS, NRAS, and KRAS (21).

A growing body of evidence indicates that elevated levels of ROS play a critical role in promoting and maintaining the malignant biological characteristics of cancer cells, particularly their aggressive metastatic phenotype (22). In the context of malignant transformation, early-stage tumor cells frequently invade adjacent stromal cells through the process of epithelial-mesenchymal transition (EMT) (23, 24). During this transition, ROS facilitate tumor metastasis by inducing Rho family GTPase-dependent cytoskeletal rearrangements, promoting the degradation of extracellular matrix proteins via matrix metalloproteinases, and enhancing angiogenesis through hypoxia-inducible factor activation (25). Additionally, various redox-regulated factors, including heat shock factor 1 (HSF1) (26), nuclear factor kappa-light-chain-enhancer of activated B cells (NF-κB) (27), and matrix metalloproteinases (MMPs) (28), also contribute to the promotion of metastasis. For instance, elevated ROS levels in tumor cells activate NF-κB, which in turn stimulates the expression of the transcription factor Snail, downregulates epithelial cadherin (E-cadherin), and increases the expression of neurocalcin and vimentin. This cascade of events disrupts intercellular connections and initiates the EMT process, thereby enhancing the metastatic potential of tumor cells (29).

Recently, a prospective study (30) conducted by Gu et al. involving 98,395 adults in the United States found that a higher OBS is associated with a reduced risk of colorectal cancer in women, however, this association was not observed in men. This finding suggests that adherence to an antioxidant-rich diet and lifestyle may contribute to the prevention of colorectal cancer. Additionally, research in a biracial US cohort (11) demonstrated that, after controlling for various confounding factors, a higher OBS reflects a predominant exposure to antioxidants, which is significantly correlated with decreased cancer mortality. Sensitivity analyses indicated that participants in the highest quartile of OBS were more likely to be non-smokers and non-drinkers. This observation suggests that individuals with higher OBS may possess greater health awareness and improved living conditions; however, the study did not account for confounding variables such as education level and household income, which may undermine the credibility of the findings. Furthermore, a recent multinational cohort study (31) examined the relationship between five lifestyle factors—body mass index (BMI), smoking, alcohol consumption, dietary habits (as measured by the 2015 Healthy Eating Index, HEI-2015), and physical activity—and mortality among cancer survivors. The results indicated that each healthy lifestyle factor—specifically, non-smoking, moderate alcohol consumption, adequate physical exercise, a nutritious diet, and optimal BMI—was independently associated with prolonged survival in cancer survivors. These findings are consistent with our own results, which reveal a negative correlation between overall OBS and the risk of mortality in cancer survivors. Similar effects were noted for independent dietary OBS and lifestyle OBS, underscoring the potential protective role of an antioxidant-rich diet and lifestyle for cancer survivors.

In summary, OBS evaluates the extent of oxidative stress by considering the interplay between various pro-oxidants and antioxidants. Oxidative stress is a critical factor in tumorigenesis, progression, and metastasis, operating through several mechanisms, such as the induction of DNA damage, the activation of oncogenic signaling pathways, the promotion of EMT, and the regulation of redox-sensitive factors. Elevated levels of ROS not only facilitate the malignant transformation of tumors but also sustain their invasive and metastatic capabilities. A high OBS is typically correlated with a more favorable prognosis in cancer patients.

Our research presents several advantages. Firstly, by employing a comprehensive scoring system (OBS) that simultaneously evaluates dietary and lifestyle factors, we conducted an extensive assessment of both pro-oxidative and anti-oxidative exposures, rather than relying on a singular indicator. This methodology facilitates a more nuanced understanding of the relationship between oxidative stress and mortality among cancer survivors. Secondly, the use of a nationally representative, high-quality sample enhances the generalizability of our findings. However, this study is not without its limitations. First, it is important to note that the cancer data utilized from the NHANES database predominantly relied on participants’ self-reported information and 24-hour dietary recall, which may have heightened the risk of diagnostic bias and potential recall bias. Second, although adjustments were made for tumor-related factors, such as since first cancer diagnosis, NHANES database lacks comprehensive data regarding the staging and treatment of specific cancers. Third, the calculation of the Oxidative Balance Score is predicated on the assumption that all oxidants and antioxidants exhibit a linear correlation with the level of oxidative stress, failing to consider the potential threshold effect of antioxidants. It is plausible that high doses of antioxidants may induce toxic pro-oxidant activity due to this threshold effect. Finally, despite our efforts to account for several confounding variables, the possibility of unrecognized confounders in our analyses and subgroup analyses cannot be entirely ruled out.

Future research plans should prioritize large-scale, multicenter randomized controlled trials to rigorously assess the effectiveness of interventions aimed at improving oxidative balance scores on survival outcomes for cancer survivors. Additionally, there is an urgent need for more comprehensive *in vitro* mechanistic studies to validate the potential mechanisms of various antioxidant therapies in this population. These efforts will provide stronger guidance for clinical practice and advance our understanding of cancer management.

## 5 Conclusion

In conclusion, the present study showed a significant negative correlation between OBS and both all-cause mortality and cancer-specific mortality among cancer survivors, utilizing data from NHANES. Specifically, higher OBS scores, indicating increased exposure to antioxidant factors relative to pro-oxidant factors in diet and lifestyle, were linked to lower risks of all-cause and cancer-specific mortality. Similar associations were noted when the dietary and lifestyle components of the OBS were analyzed separately. These findings suggest that the OBS may offer further insights into the prognostic impact of lifestyle interventions on survival rates among cancer survivors.

## Data Availability

The original contributions presented in the study are included in the article/**Supplementary Material**. Further inquiries can be directed to the corresponding author.
