# Supplementary material for: Association between oxidative balance score and all-cause and cancer-specific mortality among cancer survivors
Source: Front Immunol. 2025 Mar 20;16:1541675. doi: 10.3389/fimmu.2025.1541675 (PMC11965111; doi:10.3389/fimmu.2025.1541675)
Supplement: Supplementary file 1 [file Table1.pdf]

Supplementary Table 1: Components of the oxidative balance score

OBS components	Property	Male			Female		
		0	1	2	0	1	2
Dietary OBS components							
Dietary fiber (g/d)	A	<12.56	12.56-19.70	≥19.70	<10.10	10.10-16.31	≥16.31
Carotene (RE/d)	A	<98.83	98.83-306.25	≥306.25	<98.08	98.08-383.50	≥383.50
Riboflavin (mg/d)	A	<1.79	1.79-2.69	≥2.69	<1.34	1.34-2.02	≥2.02
Niacin (mg/d)	A	<20.65	20.65-29.75	≥29.75	<14.52	14.52-21.86	≥21.86
Vitamin B_6 (mg/d)	A	<1.59	1.59-2.40	≥2.40	<1.13	1.13-1.77	≥1.77
Total folate (mcg/d)	A	<316.00	316.00-492.00	≥492.00	<251.00	251.00-388.96	≥388.96
Vitamin B_{12} (mcg/d)	A	<3.36	3.36-6.20	≥6.20	<2.22	2.22-4.22	≥4.22
Vitamin C (mg/d)	A	<42.44	42.44-113.21	≥113.21	<38.01	38.01-98.49	≥98.49
Vitamin E (ATE) (mg/d)	A	<5.82	5.82-9.42	≥9.42	<4.53	4.53-7.52	≥7.52
Calcium (mg/d)	A	<646.00	646.00-1072.00	≥1072.00	<499.24	499.24-849.00	≥849.00
Magnesium (mg/d)	A	<257.00	257.00-361.28	≥361.28	<187.00	187.00-283.43	≥283.43
Zinc (mg/d)	A	<9.75	9.75-15.10	≥15.10	<6.73	6.73-10.75	≥10.75
Copper (mg/d)	A	<1.12	1.12-1.57	≥1.57	<0.85	0.85-1.28	≥1.28
Selenium (mcg/d)	A	<94.94	94.94-141.80	≥141.80	<67.79	67.79-99.50	≥99.50
Total fat (g/d)	P	≥69.83	69.83-107.43	<107.43	≥50.98	50.98-75.79	<75.79
Iron (mg/d)	P	≥12.88	12.88-19.17	<19.17	≥9.65	9.65-14.32	<14.32
Lifestyle OBS components							
Physical activity (MET-minute/week)	A	<400	400-1000	≥1000	<400	400-1000	≥1000
Alcohol (g/d)	P	≥30	0-30	None	≥15	0-15	None

OBS components	Property	Male			Female		
		0	1	2	0	1	2
Body mass index (kg/m^2)	P	≥25.54	25.54-29.17	<29.17	≥23.74	23.74-28.64	<28.64
Cotinine (ng/mL)	P	≥0.038	0.038-1.13	<1.13	≥0.035	0.035-0.172	<0.172

OBS: oxidative balance score; A: antioxidant; P: prooxidant; RE: retinol equivalent; ATE: alpha-tocopherol equivalent; MET: metabolic equivalent.

Supplementary Table 2. Numbers of cancer survivors by cancer type, NHANES 1999-2018.

Characteristic	total(n=4099)	Q1(n=1146)	Q2(n=1068)	Q3(n=863)	Q4(n=1022)	P value
Type of cancer,n(%)						< 0.0001
Bladder	92(1.63)	24(1.12)	31(2.61)	17(1.36)	20(1.35)	
Breast	610(14.80)	165(13.81)	147(13.61)	137(14.24)	161(17.20)	
Cervix	272(7.84)	96(11.16)	81(9.32)	47(6.47)	48(4.67)	
Colon	270(4.96)	94(5.24)	74(5.71)	55(6.19)	47(3.08)	
Kidney	76(1.37)	31(2.40)	23(1.41)	8(0.60)	14(1.02)	
Leukemia	39(1.04)	10(1.19)	15(1.40)	6(0.86)	8(0.71)	
Lung	96(1.97)	41(3.87)	22(1.44)	15(1.34)	18(1.32)	
Lymphoma/Hodgkin's disease	89(2.19)	22(2.01)	28(2.85)	19(2.47)	20(1.49)	
Melanoma	239(7.31)	53(6.98)	52(5.62)	52(7.66)	82(8.88)	
Skin (non-melanoma)	631(20.60)	124(14.24)	150(19.31)	157(23.24)	200(25.20)	
Other	405(10.10)	123(12.38)	92(9.36)	79(8.25)	111(10.27)	
Ovary	93(1.89)	33(3.23)	22(1.69)	18(1.36)	20(1.32)	
Prostate	626(9.40)	193(9.98)	171(8.77)	128(10.43)	134(8.72)	
Skin (don't know what kind)	320(9.06)	75(6.48)	87(10.15)	72(9.83)	86(9.65)	
Thyroid	80(2.05)	18(1.76)	25(2.39)	17(1.96)	20(2.05)	
Uterus	161(3.81)	44(4.16)	48(4.36)	36(3.73)	33(3.06)	

Supplementary Table 3. Multivariable Cox regression models analysis of the relationship between OBS and mortality among cancer survivors in NHANES 1999–2018.

Exposure	Model 1	Model 2	Model 3
	HR (95 % CI) P value	HR (95 % CI) P value	HR (95 % CI) P value
**All causes**			
OBS	0.972(0.959,0.984)<0.0001	0.962(0.951,0.974)<0.0001	0.976(0.964,0.988)<0.001
OBS (Quartile)			
Q1	Reference	Reference	Reference
Q2	0.720(0.574,0.904)0.005	0.686(0.566,0.833)<0.001	0.786(0.648,0.953)0.014
Q3	0.667(0.505,0.879)0.004	0.584(0.457,0.746)<0.0001	0.680(0.533,0.867)0.002
Q4	0.565(0.443,0.721)<0.0001	0.506(0.400,0.640)<0.0001	0.658(0.513,0.843) <0.001
P for Trend	<0.0001	<0.0001	<0.001
OBS.DIETARY	0.973(0.962,0.986)<0.0001	0.969(0.956,0.981)<0.0001	0.980(0.968,0.993)0.003
OBS.DIETARY(Quartile)			
Q1	Reference	Reference	Reference
Q2	0.736(0.595,0.911) 0.005	0.726(0.588,0.896)0.003	0.828(0.671,1.021)0.078
Q3	0.663(0.515,0.854) 0.001	0.578(0.461,0.726)<0.0001	0.659(0.526,0.825) <0.001
Q4	0.615(0.489,0.775)<0.0001	0.570(0.459,0.708)<0.0001	0.720(0.571,0.906)0.005
P for Trend	<0.001	<0.0001	0.001
OBS.LIFESTYLE	0.903(0.852,0.957)<0.001	0.818(0.776,0.864)<0.0001	0.870(0.819,0.923)<0.0001
OBS.LIFESTYLE(Quartile)			
Q1	Reference	Reference	Reference
Q2	0.740(0.590,0.928)0.009	0.657(0.536,0.805)<0.0001	0.738(0.598,0.911)0.005
Q3	0.767(0.601,0.980)0.034	0.622(0.499,0.774)<0.0001	0.717(0.570,0.902)0.004
Q4	0.626(0.480,0.817)<0.001	0.429(0.340,0.542)<0.0001	0.570(0.446,0.728)<0.0001
P for Trend	<0.001	<0.0001	<0.0001
**Cancer**			
OBS	0.965(0.943,0.987)0.002	0.955(0.934,0.976)<0.0001	0.964(0.944,0.985) <0.001
OBS (Quartile)			
Q1	Reference	Reference	Reference
Q2	0.645(0.453,0.919)0.015	0.602(0.424,0.853)0.004	0.672(0.477,0.947)0.023
Q3	0.558(0.381,0.818)0.003	0.486(0.332,0.712) <0.001	0.545(0.372,0.798)0.002
Q4	0.526(0.343,0.806)0.003	0.444(0.295,0.668)<0.0001	0.530(0.353,0.794)0.002
P for Trend	0.003	<0.0001	0.001

OBS.DIETARY	0.968(0.944,0.992)0.008	0.962(0.939,0.985)0.001	0.971(0.949,0.993)0.012
OBS.DIETARY(Quartile)			
Q1	Reference	Reference	Reference
Q2	0.644(0.461,0.902)0.010	0.639(0.459,0.889)0.008	0.731(0.527,1.016)0.062
Q3	0.526(0.355,0.778)0.001	0.457(0.311,0.671)<0.0001	0.500(0.337,0.741) <0.001
Q4	0.540(0.357,0.817)0.004	0.487(0.327,0.725)<0.01	0.585(0.392,0.873)0.009
P for Trend	0.002	<0.001	0.002
OBS.LIFESTYLE	0.863(0.783,0.951)0.003	0.779(0.712,0.854)<0.0001	0.805(0.729,0.890)<0.0001
OBS.LIFESTYLE(Quartile)			
Q1	Reference	Reference	Reference
Q2	0.654(0.443,0.965)0.032	0.576(0.392,0.846)0.005	0.608(0.410,0.902)0.013
Q3	0.681(0.443,1.045)0.079	0.565(0.381,0.837)0.004	0.606(0.408,0.901)0.013
Q4	0.551(0.350,0.868)0.010	0.356(0.234,0.543)<0.0001	0.424(0.273,0.667)<0.001
P for Trend	0.005	<0.0001	<0.0001

Model 1, unadjusted;

Model 2, adjusted for age, gender, race;

Model 3, adjusted for age, gender, race, education, marital status, poverty to income ratio, hypertension, diabetes, and CVD.

Supplementary Table 4. Multivariable Cox regression models analysis of the relationship between OBS and mortality among cancer survivors in NHANES 1999–2018.

**Exposure**	Model 1<br>HR (95 % CI) P value	Model 2<br>HR (95 % CI) P value	Multivariable adjusted<br>HR (95 % CI) P value
**All causes**			
OBS	0.973(0.963,0.982)<0.0001	0.966(0.957,0.975)<0.0001	0.981(0.971,0.991)<0.001
OBS (Quartile)			
Q1	Reference	Reference	Reference
Q2	0.710(0.585,0.862) <0.001	0.699(0.592,0.825)<0.0001	0.802(0.677,0.950)0.010
Q3	0.736(0.592,0.916)0.006	0.657(0.541,0.798)<0.0001	0.753(0.621,0.914)0.004
Q4	0.555(0.458,0.672)<0.0001	0.539(0.453,0.642)<0.0001	0.699(0.578,0.846)<0.001

P for Trend	<0.0001	<0.0001	<0.001
OBS.DIETARY	0.973(0.963,0.984)<0.0001	0.973(0.963,0.982)<0.0001	0.985(0.975,0.995)0.004
OBS.DIETARY(Quartile)			
Q1	Reference	Reference	Reference
Q2	0.703(0.578,0.854) <0.001	0.726(0.596,0.884)0.001	0.825(0.677,1.005)0.056
Q3	0.716(0.587,0.872) <0.001	0.656(0.546,0.788)<0.0001	0.742(0.621,0.887)0.001
Q4	0.594(0.489,0.722)<0.0001	0.603(0.505,0.718)<0.0001	0.760(0.629,0.918)0.004
P for Trend	<0.0001	<0.0001	0.001
OBS.LIFESTYLE	0.915(0.873,0.958)<0.001	0.895(0.802,0.883)<0.0001	0.891(0.847,0.937)<0.0001
OBS.LIFESTYLE(Quartile)			
Q1	Reference	Reference	Reference
Q2	0.855(0.719,1.017)0.077	0.743(0.633,0.873)<0.001	0.835(0.710,0.981)0.029
Q3	0.848(0.689,1.044)0.120	0.697(0.578,0.839)<0.001	0.799(0.657,0.971)0.024
Q4	0.621(0.493,0.782)<0.0001	0.457(0.375,0.556)<0.0001	0.595(0.485,0.730)<0.0001
P for Trend	<0.001	<0.0001	<0.0001
**Cancer**			
OBS	0.968(0.950,0.986)<0.001	0.962(0.945,0.980)<0.0001	0.973(0.957,0.990)0.001
OBS (Quartile)			
Q1	Reference	Reference	Reference
Q2	0.620(0.459,0.839)0.002	0.608(0.456,0.810)<0.001	0.653(0.491,0.868)0.003
Q3	0.648(0.469,0.895)0.008	0.576(0.416,0.798)<0.001	0.629(0.454,0.871)0.005
Q4	0.529(0.375,0.748)<0.001	0.499(0.362,0.690)<0.0001	0.604(0.442,0.825)0.002
P for Trend	<0.001	<0.0001	0.002
OBS.DIETARY	0.971(0.952,0.990)0.004	0.970(0.952,0.989)0.002	0.981(0.963,0.999)0.035
OBS.DIETARY(Quartile)			
Q1	Reference	Reference	Reference
Q2	0.610(0.453,0.820)0.001	0.625(0.466,0.838)0.002	0.681(0.509,0.911)0.010
Q3	0.602(0.445,0.812)<0.001	0.552(0.407,0.747)<0.001	0.612(0.455,0.822)0.001
Q4	0.543(0.379,0.778)0<0.001	0.538(0.383,0.756)<0.001	0.637(0.455,0.893)0.009
P for Trend	0.001	<0.001	0.006
OBS.LIFESTYLE	0.872(0.811,0.938)<0.001	0.806(0.752,0.864)<0.0001	0.840(0.780,0.905)<0.0001
OBS.LIFESTYLE(Quartile)			

Q1	Reference	Reference	Reference
Q2	0.836(0.619,1.130)0.244	0.728(0.538,0.986)0.040	0.801(0.587,1.092)0.161
Q3	0.758(0.537,1.071)0.116	0.653(0.474,0.899)0.009	0.710(0.513,0.982)0.038
Q4	0.484(0.334,0.700)<0.001	0.354(0.249,0.504)<0.0001	0.435(0.302,0.626)<0.0001
P for Trend	<0.001	<0.0001	<0.0001

Model 1, unadjusted;

Model 2, adjusted for age, gender, race;

Multivariable adjusted Cox proportional hazards regression models, adjusted for age, gender, race, education, marital status, poverty to income ratio, hypertension, diabetes, CVD, and years since the first cancer diagnosis.
